# The Prognostic Significance of Pure Ground Glass Opacities in Lung Cancer Computed Tomographic Images

**DOI:** 10.7150/jca.33132

**Published:** 2019-11-17

**Authors:** Cuicui Huang, Chao Wang, Yadong Wang, Jichang Liu, Fenglong Bie, Yu Wang, Jiajun Du

**Affiliations:** 1 Institute of Oncology, Shandong Provincial Hospital Affiliated to Shandong University, Shandong University, Jinan, 250021, China;; 2 Department of Respiratory Medicine, Shandong Provincial Qianfoshan Hospital, Jinan 250014, Shandong Province, China;; 3 Department of Thoracic Surgery, Shandong Provincial Hospital Affiliated to Shandong University, Shandong University, Jinan, 250021, China.

**Keywords:** GGO, lung cancer, prognosis, CT, adenocarcinoma

## Abstract

**Objective:** Pure ground-glass opacity (GGO) nodules have been detected with increasing frequency using computed tomography (CT). We performed a retrospective study to clarify whether lung cancer patient prognoses correlated with pure GGO nodules. We also analyzed the clinical characters of patients with pure GGO nodules to provide diagnostic guidance on lung cancer identification and treatment of patients in clinical practice.

**Methods:** We enrolled 39 of 1422 patients with pure GGO nodules who accepted surgical treatment of the lung cancer nodules, and reviewed materials from 404 patients to verify our conclusions. To discover which factors were prognostically significant, we used the Kaplan-Meier method to estimate the overall survival (OS) and progression-free survival (PFS) curves. Age, gender, smoking history, histology, tumor size, and stage were the factors examined in our study. We also performed subgroup and matching group analyses to clarify the correlation between the presence of pure GGO nodules and prognoses.

**Results:** Pure GGO nodules were associated with non-smoking females that had adenocarcinoma. The prognoses of patients in the pure GGO nodule group was better than those in the non-pure GGO nodule group (p = 0.046). Age, grade, and stage (including tumor size and lymph node metastases) were had prognostic significance. In the matching group stage assessments, although patient prognoses were not significantly different among patients of the GGO group compared with thoses of the other group in long-term, while in the short term, patients with pure GGO nodules had longer PFS. Non-smoking female patients with lung cancer were more likely to have adenocarcinoma.

**Conclusions:** As a subgroup of GGO nodules, pure GGO nodules predict a better prognosis in all lung cancer patients. Wheras our study showed that lung patients with pure GGO nodules in similar stages were not significantly different in long-term prognoses, in the short term; patients with pure GGO nodules had longer PFS.

## Introduction

Lung cancer is a leading cause of death worldwide and imparts a heavy burden on the economies of both more developed and less developed countries. In 2018, 13% of male and female cancer patients were diagnosed with lung cancer for the first time, and the occurrence of lung cancer continues to increase 1. A previous article by the National Lung Screening Trial reported that computed tomography (CT) screening could decrease the mortality associated with lung cancer and strongly supported the use of low-dose helical CT in clinical screenings 2.

Pure ground-glass opacity (GGO) nodules are important indicators of lung cancer on CT. GGO nodules are defined as hazy areas, which do not block the parenchymal structures, vessels, and airways under the nodules 3, 4. GGO nodules are divided into two categories according to the different solid component percentages: 1) Pure GGO nodules with no solid component within the nodules and 2) Partially solid nodules with both GGO and solid components 4. GGO nodules are not a specific manifestation of lung cancer and can also indicate other lung pathologies such as hemorrhage, inflammation, and fibrosis 5. Several studies have reported that GGO nodules could be closely related to lung cancer prognoses. Moreover, mixed GGO nodules are considered to be more invasive when compared with pure GGO nodules 6-11. Saji et al. proposed that the only solid nodular components other than whole tumor sizes detected with high-resolution computed tomography were associate with prognoses and malignancies 12. The relationship of GGO nodules with prognoses remains controversial in articles published in the last decade. The association between pure GGO nodules and prognoses has been underreported, and the number of patients with pure GGO nodules in previous retrospective studies was mostly limited. Pure GGO nodules should be given more attention as an important category of GGO nodules.

In this article, we performed a retrospective study to clarify whether pure GGO nodules correlated with prognoses in lung cancer patients. We also analyzed the clinical characteristics of patients with pure GGO nodules to provide information regarding lung cancer diagnoses and treatments for patients in clinical practice.

## Methods

### Patients

This study was approved by the Ethics Committee of the Provincial Hospital Affiliated with Shandong University, Shandong University, Jinan, China. Every patient in this retrospective study was provided with and read the written informed consent during hospitalization. We reviewed the materials of 1470 patients who had accepted lung cancer resection at our institution between January 2008 and June 2014 and enrolled 1422 patients in this study based on the following criteria: 1) the resected tumor was diagnosed on histopathology as lung cancer; 2) patients had underwent CT scans in our hospital for which a complete diagnostic report was found; 3) patients had a single lesion; 4) patients did not suffer from other malignant cancers except for lung cancer; 5) prior to surgery, patient did not receive other lung surgeries, chemotherapies, or radiotherapies; 6) patients did not have stage Ⅳ lung cancer; and 7) follow-up data were available. To clarify the correlation between pure GGO nodules and prognoses, patients with pure GGO nodules were selected for analysis in our study. We also reviewed materials from 404 patients who had accepted lung cancer resection at our institution between July 2014 and March 2015 to verify our conclusions. The selection criteria were the same as noted for the initial study.

### Measurements

The basic information covered in our retrospective study included age, gender, and smoking status of patients collected from the admissions records. Doctors evaluated the possibility of resection according to the tumor stage, which was estimated by chest radiography and physical examination. The status of lymph nodes was determined using histopathologic examination. After the surgeries, patients were followed to get health status information each year until death or lost to follow-up. Once a recurrence and metastasis occurred, CT screening frequencies changed from every year to every half year. All CT images were read by more than two experienced doctors independently. Nodules were repeatedly measured to acquire the average diameters.

### Statistical Analysis

In this retrospective study, we used the Kaplan-Meier method to estimate the overall survival (OS) and progression-free survival (PFS) curves and used the log-rank test to make comparisons. Hazard ratios (HRs), 95% confidence intervals (CIs) were performed, and Cox regression analyses were used to determine if the clinical variables were prognostically significant. A two-sided p-value <0.05 indicated that a variable was prognostically significant. All data were analyzed by SPSS software (version 19, SPSS Inc., Chicago, IL, USA).

Compared with other lung cancer patients in our study, the number of patients with pure GGO nodules was still relatively small. To comprehensively analyze the prognostic significance of pure GGO nodules, we performed individual matching (1:2) by size and stage, respectively. For the size-matching group, the matching characteristics were size, age, gender, and smoking status. For the stage-matching group, the matching characteristics were tumor stage, age, gender, and smoking status. We also performed multivariate analyses on data from matched pairs of 41 patients with pure GGO nodules and 82 patients without pure GGO nodules. Moreover, subgroup analysis was also an important part of our study. We divided the enrolled patients into subgroups according to their smoking status, gender, age, pathologic diagnosis. The Kaplan-Meier method was used to study the influence of the subgroups on prognoses.

## Results

### Pure GGO nodules were associated with no-smoking females with adenocarcinoma

We divided 1422 lung cancer patients who received surgery at our hospital into two groups according to whether patients had pure GGO nodules. After surgery, the median follow-up period was 45.0 months (1-100). Table 1 shows the clinical characteristics of 39 lung cancer patients with pure GGO nodules and 1383 patients without pure GGO nodules. There was no significant difference between the two groups for age (p=0.197). While we revealed that non-pure GGO nodules were associated with males (p=0.001), smoking (p<0.001), and lymph node metastases (p<0.001), we also found that most pure GGO nodules were diagnosed at earlier stage (p<0.001) and the size of nodules was smaller (p<0.001) than the other nodule types. Most pure GGO nodules were confirmed to be well-differentiated as pathologically diagnosed (p<0.001). And adenocarcinoma is the most common histology among patients with pure GGO nodules (p<0.001). Only one case in our study was found lymph node metastasis.

### Pure GGO nodules were associated with a better prognosis compared with non-pure GGO nodules

**Figure 1:**
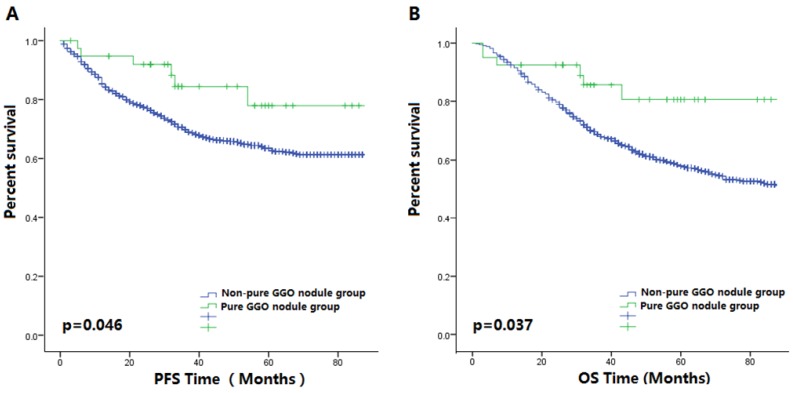
** (A)** The Kaplan-Meier survival curve for PFS between 41 patients with pure GGO nodules and 1390 patients with other lung cancer nodules after surgery (P-value = 0.046).** (B)** The Kaplan-Meier survival curve for OS between 41 patients with pure GGO nodules and 1390 patients with other lung cancer nodules after surgery (P-value = 0.037).

Figure 1 shows the survival curves of the two groups. Kaplan-Meier analysis demonstrated that pure GGO nodules were significantly associated with longer PFS (p=0.046) and OS (p=0.037) in lung cancer patients, which was similar to that seen with our verification process supplementary figure 1A-B), and the clinical information was shown in Supplementary Table 1. To show the effect of pure GGO nodules on OS and PFS more clearly, we performed univariate analysis of common clinicopathologic factors for prognosis. Results in Table 2 showed smaller tumor sizes, less invasion into lymph nodes, more-differentiated tumors, and earlier stages at diagnosis were favorable predictors for PFS in lung cancer patients. A multivariate Cox model that adjusted for the pure GGO nodules, tumor sizes, lymph node invasiveness, and tumor grades and stages was performed. Our results showed that the size (HR=1.281, 95%CI 1.051-1.562, p=0.014), lymph node metastatic rate (HR=1.670, 95%CI 1.299-2.146, p<0.001), tumor grade (p=0.001), tumor stage (p=0.006) were independent prognostic factor for OS.

### There were no significant differences in the prognoses of patients with pure GGO nodules compared with those with non-pure GGO nodules regarding tumor stage matching analyses

**Figure 2:**
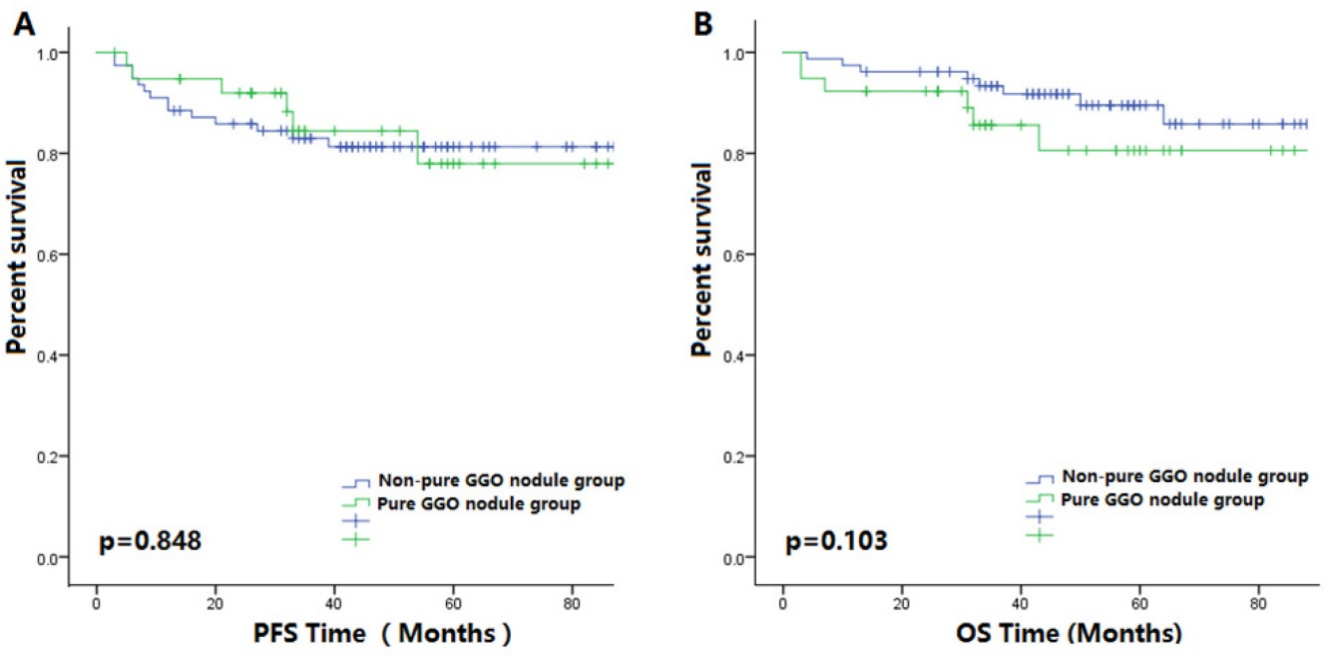
** (A)** The Kaplan-Meier survival curve for PFS between 41 patients with pure GGO nodules and 82 patients with matching tumors stages after surgery (P value is 0.848). **(B)** The Kaplan-Meier survival curve for OS between 41 patients with pure GGO nodules and 82 patients with matching tumor stages after surgery (P-value is 0.103).

In the stage matching group, clinical characteristics of the pure GGO nodule group and the non-pure GGO nodule group were similar, as shown in Table 3. Kaplan-Meier analyses showed that no significant differences in patient prognoses were observed in patients with pure GGO nodules compared with those that had non-pure GGO nodules (Figure 2. In the subgroup analyses, the age and gender subgroups showed no differences between the pure GGO nodule and non-pure GGO nodule groups for prognoses.

In our verification process, results were different, Kaplan-Meier analyses showed that there were no significant differences in the OS (p=0.375, supplementary figure 1D) of patients with pure GGO nodules and those with non-pure GGO nodules. Regarding PFS, we found that patients with pure GGO nodules achieved a longer PFS (p=0.016, supplementary figure 1C). The clinical information was shown in Supplementary Table 2. These two results could be caused by different follow-up times. In the stage matching group, the pure GGO nodule group had a higher PFS during short-term follow-up, but there was no significant difference in OS.

### Non-smoking female patients with lung cancer were more likely to have adenocarcinoma

**Figure 3:**
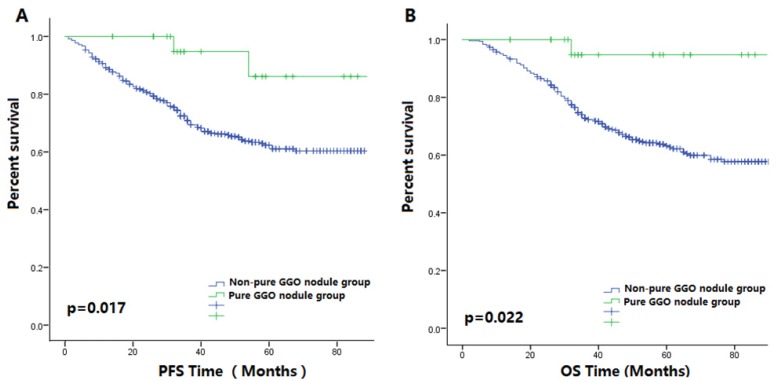
** (A)** The Kaplan-Meier survival curve for PFS between patients with pure GGO nodules and patients with other lung cancer nodules after surgery in the non-smoking subgroup (P-value = 0.017). **(B)** The Kaplan-Meier survival curve for OS between patients with pure GGO nodules and patients with other lung cancer nodules after surgery in the non-smoking subgroup (P-value = 0.022).

**Figure 4:**
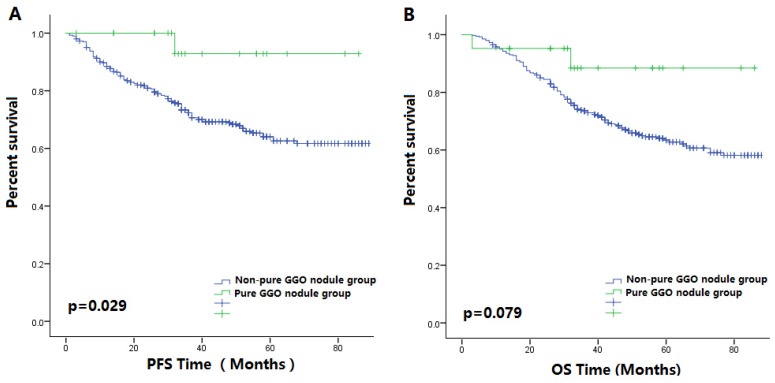
** (A)** The Kaplan-Meier survival curve for PFS between patients with pure GGO nodules and patients with other lung cancer nodules after surgery in the female subgroup (P value = 0.029). **(B)** The Kaplan-Meier survival curve for OS between patients with pure GGO nodules and patients with other lung cancer nodules after surgery in the female subgroup (P value = 0.076).

Figure 3A-B showed that in the non-smoking subgroup, patient prognoses in pure GGO nodule group was better than those in the non-pure GGO nodule group. In the female patient subgroup, the PFS of patients with non-pure GGO nodules was shorter compared with those with pure GGO nodules (p=0.029, Figure 4A-B). Among the female non-smoking subgroup, the histology of pure GGO nodules and non-pure GGO nodules was similar, and most of those patients were diagnosed as adenocarcinoma. However, in this subgroup, most pure GGO nodules were diagnosed at earlier stages (p<0.001), had a lower pathologic grade (p<0.001), smaller nodules (p=0.006), and no lymph node metastases compared with the non-pure GGO nodules.

## Discussion

GGO nodules are an important CT manifestation found in clinical practice. Pure GGO nodules were thought to indicate well-differentiated lung tumors and predict good prognoses in some previous case reports. In our study, most of pure GGO nodules were found in the early stages of the disease, and there was less lymph node invasion compared with other lung cancer nodules. The prognoses were similar between lung cancer patients with pure and non-pure GGO nodules, as previously reported. It has been shown that pure GGO nodules took longer to double in size compared with mixed GGO nodules and solid nodules 13, 14. Park et al. reported no recurrences or metastases after 24 months in a patient who had a pure GGO nodule resected. There was also no significant difference in the prognoses in terms of tumor size 15. However, our study showed that tumor size played an important role in prognoses. Because most lung cancer patients with pure GGO nodules were in earlier disease stages, and had smaller tumor sizes compared with those without pure GGO nodule, both earlier disease stages and smaller tumor sizes are thought to predict a better prognosis as reported in a previous article 16. Therefore, follow-up periods should be longer when wanting to clarify the correlation between pure GGO nodules and prognosis. The follow-up time in our study was 100 months, which was five times longer than that of the study by Park et al.

Takatoshi Aoki reported that about 10-25% of pure GGO nodules increase in size or gradually become solid nodules 17. Although, there was a published article which described invasiveness and malignant potential of pulmonary lesions presenting as pure GGO nodules and reported 12% of examined patients with pure GGO were diagnosed as invasive cancers. Besides, it was commonly considered that the pure GGO nodules were more stable than those with mixed GGO nodules 18. However, our results showed that prognosis of patients with pure GGO nodules was not as good as we had expected in the long-term follow-up stage matching study; there was no significant difference in the tumor stages between pure GGO nodule group and non-pure GGO nodule group. While at the short-term follow-up visits, the pure GGO nodule group had a higher PFS. Although there was no difference in long-term prognoses in the stage matching study, in the short-term, the pure GGO nodule group had a longer PFS compared with the non-pure GGO nodule group. One hypothesis was that GGO nodules were not specific signs for lung cancer. The opacity in the CT images increased because cells and fluid-filled part of alveolar lumen and air was partially decreased 4. Thus GGO nodules also can indicate another lung disease such as inflammation, fibrosis, and edema 13. Inflammation was also thought to play an important role in the occurrence and development of tumors. Some markers of inflammation were confirmed to be associated with poor prognoses 19. In addition to these diseases, some GGO nodules were in unstable, which can lead to a worse prognosis. Mao et al. reported no differences were seen between the lesions that were not invasive and those that were invasive regarding shape, size, and window when the Pearson X^2^ test was used for analysis. It is still hard to distinguish the malignant nodules and well-differentiated nodules by size on CT image^20^.

The female non-smoking subgroup was the most concerning of the two subgroups in our study. Oh et al. reported that during a follow-up period, female patients with GGO nodules were at a greater risk of having a poor prognosis compared with those that did not have GGO nodules 21. Most malignant GGO nodules were diagnosed as adenocarcinoma as previous articles reported 22-24. Adenocarcinoma also accounts for a large percentage of lung tumors in female patients 25. The facts mentioned above can explain why pure GGO nodules indicate good prognoses in female patients as opposed to male patients. Smoking status is the dominant factor influencing prognosis in the univariate analysis of our study. An interesting caveat is that in the non-smoking subgroup, the prognosis of patients with pure GGOs significantly differed from that of patients without pure GGO nodules. A retrospective study looked at 5-year survival in non-smoking lung cancer patients compared with smokers and found that smoking was a predictor of an unfavorable prognosis. Although the mechanisms of how smoking influenced the prognosis of lung patients were not clear, some hypotheses were formed. Smoking was shown to impair both local and systematic immunity by reducing the actions of T and B lymphocytes, and NK cells. Moreover, smoking also can also cause peripheral blood leukocytosis 26.

There were some limitations in our study. First, because our study was retrospective, an inherent bias was unavoidable. Future prospective studies should be considered, and patient characteristics should be considered to create detailed projects that can minimize bias. Second, the number of patients with pure GGO nodules in our study was small because of the relatively low use of CT screening in the past few decades. Meanwhile, the incidence of pure GGO nodules was reasonably low when compared with solid nodules. Therefore, larger studies are warranted in the future.

In conclusion, as a subgroup of GGO nodules, pure GGO nodules predict a better prognosis in lung cancer patients. For the first time, we showed that when comparing lung patients with similar stage nodules, although there was no difference in long-term prognoses, in the short term, patients with pure GGO nodules had longer PFS. In clinical practice, it is important to consider the correlation between pure GGO nodules and improved prognoses.

## Supplementary Material

Supplementary figures and tables.

## Figures and Tables

**Table 1:** The association of pure GGO nodules with the clinicopathologic characteristics of 1422 patients with lung cancer and treated with surgery.

Characteristic	Total, n=1422	Non-pure GGO nodule, n=1383	Pure GGO nodule, n=39	p-value
**Gender, n (%)**				
Male	999 (70.3)	981 (70.9)	18 (46.2)	0.001
Female	423 (29.7)	402 (29.1)	21 (53.8)	
**Age, years**				
≤60	767 (53.9)	742 (53.7)	25 (64.1)	0.197
>60	655 (46.1)	641 (46.3)	14 (35.9)	
**Smoking history, n (%)**				
Never	518 (36.4)	492 (33.5)	26 (66.7)	<0.001
Former	178 (12.5)	175 (12.6)	3 (7.7)	
Current	726 (51.0)	716 (51.7)	10 (25.6)	
**Histology, n (%)**				
Adenocarcinoma	697 (49.0)	663 (47.9)	34 (87.1)	<0.001
Squamous	519 (36.5)	519 (37.5)	1(2.6)	
SCLC	109 (7.7)	109 (7.9)	0 (0)	
Other	97 (6.8)	92 (6.7)	4(10.3)	
**Tumor size, n (%)**				
≤3cm	663 (46.6)	635 (45.9)	34 (87.2)	<0.001
>3cm	759 (53.3)	748 (54.1)	5 (12.8)	
**Lymph node metastasis, n (%)**				
No	771 (54.2)	734(53.1)	37 (94.9)	<0.001
Yes	651 (45.8)	649(46.9)	2 (5.1)	
**Pathological grade, n (%)**				
Well	169 (11.9)	143 (10.3)	26 (66.7)	<0.001
Moderately	645 (45.4)	642 (46.4)	3 (7.7)	
Poorly	493 (34.6)	491 (35.6)	2 (5.1)	
Unknown	115 (8.1)	107 (7.7)	8 (20.5)	
**Pathological stage, n (%)**				
I	566 (39.8)	533 (38.5)	33 (84.6)	<0.001
II	392 (27.6)	388 (28.1)	4 (10.3)	
III	464 (32.6)	462 (33.4)	2 (5.1)	

SD standard deviation; SCLC small cell lung cancer; GGO ground glass opacity.

**Table 2:** Univariate and multivariate analyses of prognostic factors for OS and PFS.

	OS				PFS			
Characteristics	Univariate		Multivariate		Univariate		Multivariate	
	HR (95%CI)	p	R (95%CI)	p	HR (95%CI)	p	HR (95%CI)	p
**Pure GGO nodules**								
No	1		1		1		*	*
Yes	0.464 (0.220-0.977)	0.043	1.417 (0.649-3.094)	0.381	0.451 (0.202-1.010)	0.053	*	*
**Age**								
≤60	1		1		1		*	*
>60	1.241 (1.052-1.463)	0.010	1.372 (1.158-1.624)	<0.001	1.034 (0.860-1.244)	0.722	*	*
**Gender**								
Female	0.744 (0.616-0.899)	0.002	1.148 (0.876-1.504)	0.317	1.182 (0.964-1.449)	0.109	*	*
Male	1		1		1		*	*
**Histological subtype**								
Adenocarcinoma	1		1		1		*	*
Squamous	1.156 (0.965-1.385)	0.115	0.801 (0.655-0.979)	0.031	0.871 (0.711-1.068)	0.184	*	*
SCLC	1.598 (1.197-2.133)	0.001	0.838 (0.612-1.148)	0.270	1.137 (0.807-1.601)	0.463	*	*
Others	0.993 (0.698-1.414)	0.045	1.081 (0.656-1.780)	0.760	0.776 (0.514-1.172)	0.228	*	*
**Smoking index**								
Never	1		1		1		*	*
Former	1.512 (1.167-1.960)	0.002	1.121 (0.805-1.561)	0.500	1.306 (0.981-1.738)	0.067	*	*
Current	1.66 (1.134-1.645)	0.001	1.007 (0.772-1.314)	0.959	1.053 (0.861-1.288)	0.615	*	*
**Tumor size**								
≤3cm	1		1		1		1	
>3cm	1.909 (1.606-2.269)	<0.001	1.281 (1.051-1.562)	0.014	1.758 (1.452-2.128)	<0.001	1.278 (1.029-1.578)	0.027
**Lymph node metastasis**								
No	1		1		1		1	
Yes	2.904 (2.442-3.455)	<0.001	1.670 (1.299-2.146)	<0.001	2.297 (1.901-2.774)	<0.001	1.428 (1.082-1.884)	0.012
**Pathological TMN stage**								
Ⅰ	1		1		1		1	
II	2.416 (1.907-3.060)	<0.001	1.541 (1.135-2.090)	0.006	2.035 (1.587-2.610)	<0.001	1.345 (0.969-1.865)	0.076
III	4.219 (3.396-5.241)	<0.001	2.264 (1.615-3.174)	<0.001	3.063 (2.431-3.860)	<0.001	1.759 (1.217-2.543)	<0.001
**Differential degree**								
Well	1		1		1		1	
Moderate	3.420 (2.215-5.280)	<0.001	2.220 (1.406-3.505)	0.001	2.197 (1.480-3.261)	<0.001	1.524 (1.017-2.286)	0.041
Poor	4.302 (2.777-6.663)	<0.001	2.514 (1.580-4.002)	<0.001	3.039 (2.044-4.519)	<0.001	1.934 (1.282-2.917)	0.002
Unknown	2.527 (1.497-4.264)	0.001	1.843 (0.986-3.444)	0.055	1.641 (0.976-2.759)	0.062	1.346 (0.798-2.269)	0.226

Abbreviation: OS= overall survival; PFS=progression-free survival; HR=hazard ratio; CI=confidence interval; SCLC=small cell lung cancer.

**Table 3:** The association of pure GGO nodules with the clinicopathologic characteristics of 117 patients with individual-matching according to tumor stage.

Characteristic	Total, n=117	Non-pure GGO nodule, n=78	Pure GGO nodule, n=39	p-value
**Gender, n (%)**				
Male	54 (46.2)	36 (46.2)	18 (46.2)	1.000
Female	63 (53.8)	42 (53.8)	21 (53.8)	
**Age, years**				
≤60	77 (65.8)	52 (66.7)	25 (64.1)	0.783
>60	40 (34.2)	26 (33.3)	14 (35.9)	
**Smoking history, n (%)**				
Never	79 (67.5)	53 (67.9)	26 (66.7)	0.664
Former	6 (5.1)	3 (3.8)	3 (7.7)	
Current	32 (27.4)	22 (28.2)	10 (25.6)	
**Histology, n (%)**				
Adenocarcinoma	102(87.2)	68 (87.2)	34 (87.2)	1.000
Squamous	3(2.6)	2(2.6)	1(2.6)	
SCLC	0 (0.0)	0 (0.0)	0 (0)	
Other	12 (10.2)	8 (10.2)	4(10.2)	
**Tumor size, n (%)**				
≤3cm	96 (82.1)	62 (79.5)	34 (87.2)	0.307
>3cm	21 (17.9)	16 (10.5)	5 (12.8)	
**Lymph node metastasis, n (%)**				
No	106 (90.6)	69 (88.5)	38 (97.4)	0.102
Yes	11 (9.4)	9 (11.5)	1 (2.6)	
**Pathological grade, n (%)**				
Well	46 (39.3)	20 (25.6)	26 (66.7)	<0.001
Moderately	34 (29.1)	31 (39.7)	3 (7.7)	
Poorly	13 (11.1)	11 (14.1)	2 (5.1)	
Unknown	24 (20.5)	16(20.5)	8 (20.5)	
**Pathological stage, n (%)**				
I	99 (84.6)	66(84.6)	33 (84.6)	1.000
II	12 (10.3)	8 (10.3)	4 (10.3)	
III	6 (5.1)	4 (5.1)	2 (5.1)	

SD standard deviation; SCLC small cell lung cancer; GGO ground glass opacity.

**Table 4:** The association of pure GGO nodules with the clinicopathologic characteristics of 380 non-smoking female patients.

Characteristic	Total, n=380	No pure GGO nodule, n=361	Pure GGO nodule, n=19	p-value
**Age, years**				
≤60	235 (61.8)	223 (61.8)	12 (63.2)	0.904
>60	145 (38.2)	138 (38.2)	7 (36.8)	
**Histology, n (%)**				
Adenocarcinoma	301 (79.2)	285 (78.9)	16 (84.2)	0.764
Squamous	30 (7.9)	30 (8.3)	0(0.0)	
SCLC	23 (6.1)	23 (6.3)	0(0.0)	
Other	26 (6.8)	23 (6.3)	3(15.8)	
**Tumor size, n (%)**				
≤3cm	226(59.5)	209 (57.9)	17(89.5)	**0.006**
>3cm	154 (40.5)	152 (42.1)	2 (10.5)	
**Lymph node metastasis, n (%)**				
No	217 (57.1)	198 (54.8)	19(100.0)	**0.000**
Yes	163 (42.9)	163 (45.2)	0 (0.0)	
**Pathological grade, n (%)**				
Well	86 (22.6)	73(20.2)	13 (68.4)	**0.000**
Moderately	138 (36.3)	137 (37.9)	1 (5.3)	
Poorly	115 (30.3)	115 (31.9)	0(0.0)	
Unknown	41(10.8)	36(10.0)	5 (26.3)	
**Pathological stage, n (%)**				
I	184 (48.4)	168 (46.5)	16 (84.2)	**0.001**
II	83(21.8)	81(22.4)	2 (10.5)	
III	113 (29.7)	112 (31.0)	1(5.3)	

## Abbreviation

GGO: Ground-glass opacity
HRs: Hazard ratios
CIs: Confidence intervals
CT: Computed tomography
OS: overall survival
PFS: progression-free survival
