# Supplementary material for: The Prognostic Significance of Pure Ground Glass Opacities in Lung Cancer Computed Tomographic Images
Source: J Cancer. 2019 Nov 17;10(27):6888–95. doi: 10.7150/jca.33132 (PMC6909955; doi:10.7150/jca.33132)
Supplement: Supplementary file 1 — Supplementary figures and tables. [file jcav10p6888s1.pdf]

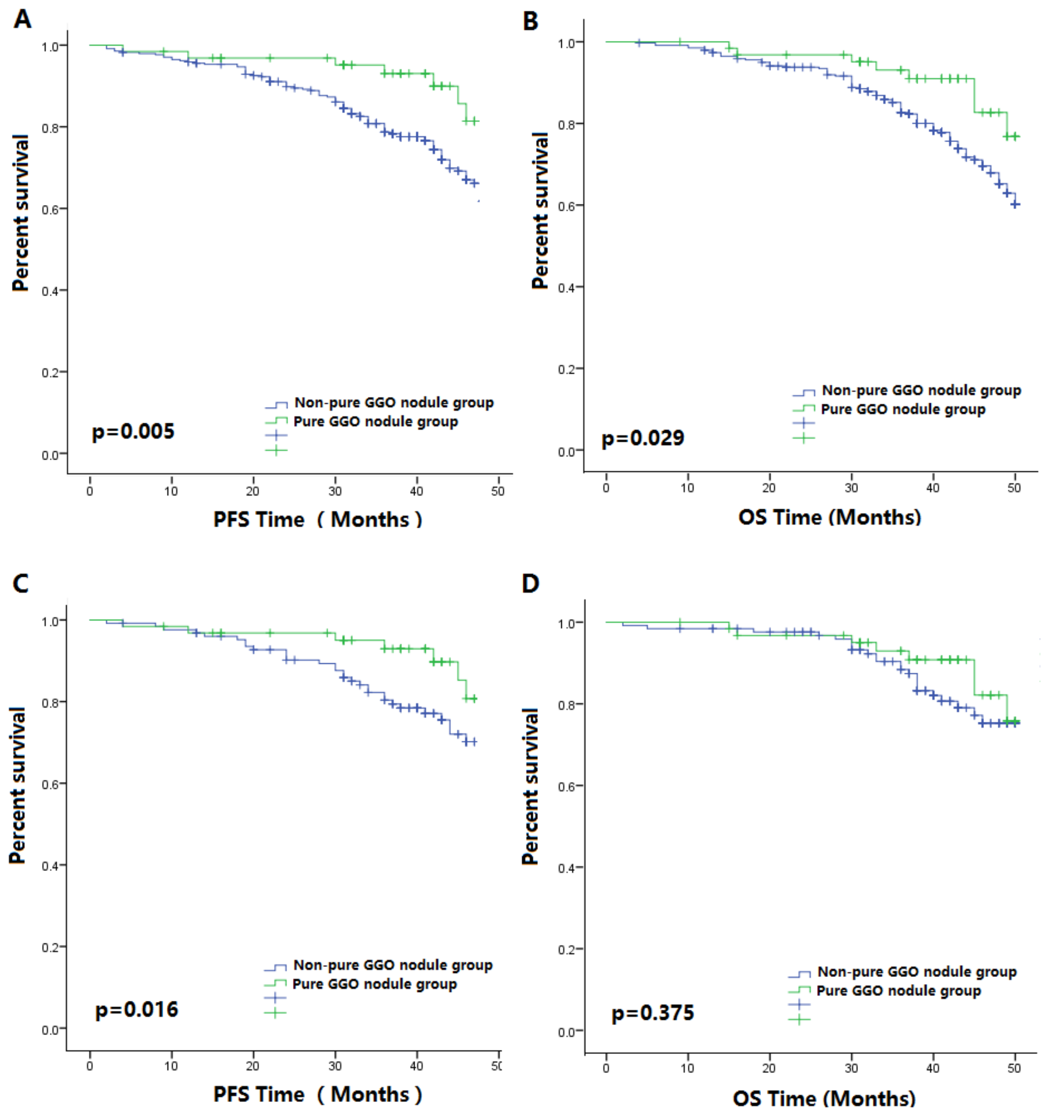

Supplementary Figure 1A. The Kaplan-Meier survival curve for PFS between 63 patients with pure GGO nodules and 341 patients with other lung cancer nodules after surgery (p-value = 0.005). B. The Kaplan-Meier survival curve for OS between 63 patients with pure GGO nodules and 341 patients with other lung cancer nodules after surgery (p-value = 0.029). C. The Kaplan-Meier survival curve for PFS between 63 patients with pure GGO nodules and 126 patients with matching tumor stages after surgery (p-value = 0.016). D. The Kaplan-Meier survival curve for OS between 63 patients with pure GGO nodules and 126 patients with matching tumor stages after surgery (p-value is 0.375).

Supplementary Table 1. The association of pure GGO nodules with the clinicopathologic characteristics of 404 patients with lung cancer and treated with surgery.

Characteristic	Total, n=404	Non-pure GGO nodule, n=341	Pure GGO nodule, n=63	p-value
**Gender, n (%)**				
Male	261 (64.6)	232 (68.0)	29 (46.0)	0.001
Female	143 (35.4)	109 (32.0)	34 (54.0)	
**Age, years**				
≤60	224 (55.4)	190 (55.7)	34 (54.0)	0.7197
＞60	180 (44.6)	151 (44.3)	29 (46.0)	
**Histology, n (%)**				
Adenocarcinoma	256 (63.4)	201 (58.9)	55 (87.3)	<0.001
Squamous	111 (27.5)	107 (31.4)	4 (6.3)	
SCLC	12 (3.0)	11 (3.2)	1 (1.6)	
Other	25 (6.1)	22 (6.5)	3(4.8)	
**Tumor size, n (%)**				
≤3cm	260 (63.4)	208 (61.0)	52 (82.5)	<0.001
＞3cm	144 (35.6)	133 (39.0)	11 (17.5)	
**Lymph node metastasis, n (%)**				
No	268 (66.3)	219(64.2)	49 (77.8)	0.036
Yes	136 (33.7)	122(35.8)	14 (22.2)	
**Pathological stage, n (%)**				
I	225 (55.7)	179 (52.5)	46 (73.0)	0.003
II	96 (23.8)	89 (26.1)	7 (11.1)	
III	83 (20.5)	73 (21.4)	10 (15.9)	

SD standard deviation; SCLC small cell lung cancer；GGO ground glass opacity

Supplementary Table 2. The association of pure GGO nodules with the clinicopathologic characteristics of 189 patients with individual-matching according to tumor stage.

Characteristic	Total, n=189	Non-pure GGO nodule, n=126	Pure GGO nodule, n=63	p-value
**Gender, n (%)**				
Male	86 (45.5)	57 (45.2)	29 (46.0)	0.918
Female	103 (54.5)	69 (54.8)	34 (54.0)	
**Age, years**				
≤60	106 (56.0)	72 (57.1)	34 (54.0)	0.678
＞60	83 (44.0)	54(42.9)	29 (46.0)	
**Histology, n (%)**				
Adenocarcinoma	165(87.3)	110 (87.3)	55 (87.3)	1.000
Squamous	12(6.3)	8(6.3)	4 (6.3)	
SCLC	3 (1.6)	3 (2.3)	1 (1.6)	
Other	9 (4.8)	5 (4.0)	3(4.8)	
**Tumor size, n (%)**				
≤3cm	147 (77.8)	95 (75.4)	52 (82.5)	0.266
＞3cm	42 (22.2)	31 (24.6)	11 (17.5)	
**Lymph node metastasis, n (%)**				
No	133 (70.4)	84 (66.7)	49 (77.8)	0.115
Yes	56 (29.6)	42 (33.3)	14 (22.2)	
**Pathological stage, n (%)**				
I	138 (73.0)	92(73.0)	46 (73.0)	1.000
II	21 (11.1)	14 (11.1)	7 (11.1)	
III	30 (15.9)	20 (15.9)	10 (15.9)	

SD standard deviation; SCLC small cell lung cancer；GGO ground glass opacity
